# High frequency of antidrug antibodies and association of random drug levels with efficacy in certolizumab pegol-treated patients with rheumatoid arthritis: results from the BRAGGSS cohort

**DOI:** 10.1136/annrheumdis-2015-208849

**Published:** 2016-05-31

**Authors:** Meghna Jani, John D Isaacs, Ann W Morgan, Anthony G Wilson, Darren Plant, Kimme L Hyrich, Hector Chinoy, Anne Barton

**Affiliations:** 1 Arthritis Research UK Centre for Genetics and Genomics, Centre for Musculoskeletal Research, Institute of Inflammation and Repair, University of Manchester, Manchester Academic Health Science Centre, Manchester, UK; 2 Arthritis Research UK Centre for Epidemiology, Centre for Musculoskeletal Research, University of Manchester, Manchester, UK; 3 Musculoskeletal Research Group, Institute of Cellular Medicine, Newcastle University and National Institute of Health Research Newcastle Biomedical Research Centre, Newcastle upon Tyne, UK; 4 Leeds Institute of Rheumatic and Musculoskeletal Medicine, University of Leeds and National Institute of Health Research Leeds Musculoskeletal Biomedical Research Unit, Leeds Teaching Hospitals NHS Trust, Leeds, UK; 5 University College of Dublin School of Medicine and Medical Science, Dublin, Ireland; 6 National Institute of Health Research Manchester Musculoskeletal Biomedical Research Unit, Central Manchester Foundation Trust, Manchester Academic Health Science, Manchester, UK

**Keywords:** DMARDs (biologic), Anti-TNF, Rheumatoid Arthritis, TNF-alpha, Treatment

## Abstract

**Objectives:**

To evaluate (i) the association between random certolizumab drug levels, antidrug antibodies (ADAbs) and treatment response in patients with rheumatoid arthritis (RA); (ii) longitudinal factors associated with ADAbs and certolizumab drug levels.

**Methods:**

This prospective cohort included 115 patients with RA treated with certolizumab. Serum samples were collected at 3, 6 and 12 months following treatment initiation. Drug levels and ADAbs were measured using ELISA and radioimmunoassay, respectively, at 3, 6 and 12 months. Disease Activity Score in 28 joints (DAS28) were measured at each visit and 12 months European League Against Rheumatism (EULAR) response was calculated. Patient self-reported adherence was collected longitudinally. Ordinal logistic regression and generalised estimating equation were used to test the association: (i) between drug levels, from serum sampled and treatment response; (ii) between ADAbs and drug levels; (iii) patient-centred factors and drug levels.

**Results:**

ADAbs were detected in 37% (42/112 patients by 12 months). The presence of ADAbs were significantly associated with lower drug levels over 12 months (β=−0.037, 95% CI −0.055 to 0.018, p<0.0001) but not independently with 12 months EULAR response (β=0.0013 (95% CI −0.0032 to 0.00061), p=0.18). Drug level was associated with 12 months EULAR response (β=0.032 (95% CI 0.0011 to 0.063), p=0.042). In the multivariate model, ADAb level and adherence were significantly associated with drug concentrations.

**Conclusions:**

This is the first study to demonstrate that higher certolizumab drug levels are associated with better 12 months EULAR response. ADAbs in certolizumab-treated patients with RA were detected at higher levels than previous studies and help determine the aetiology of a low drug level.

## Introduction

Although tumour necrosis factor inhibitor (TNFi) drugs, such as certolizumab pegol, have been shown to be efficacious in the treatment of rheumatoid arthritis (RA), not all patients respond to treatment.^1^ ^2^ In infliximab, adalimumab and golimumab-treated patients, absent/reduced treatment response may be the result of low drug concentrations due to immunogenicity (formation of antidrug antibodies (ADAbs)).^3–5^ ADAbs can reduce drug concentrations via antibody-mediated drug clearance, and reduce efficacy by preventing the drug binding to its target. Certolizumab pegol is a PEGylated Fab’ fragment of a recombinant humanised antibody directed against TNF. While PEGylation has been shown to reduce ADAbs in some proteins,^6^ it may increase it in others.^7^

Initial registration trials in certolizumab-treated patients with RA revealed low ADAb levels using an ELISA, ranging 5.1%–6.1%.^1^ ^2^ In a post hoc Crohn's disease trial analysis, higher certolizumab drug levels were associated with endoscopic response and remission.^8^ To date, there have been no prospective observational studies assessing drug levels and ADAbs for correlation with treatment response in certolizumab-treated patients with RA. The aims of this study were to (i) assess the incidence of ADAbs in certolizumab-initiated patients with RA using sensitive detection techniques; (ii) test the association between certolizumab drug levels, ADAbs and 12 months treatment response in patients with RA; (iii) assess baseline and longitudinal factors associated with certolizumab drug levels and ADAb formation.

## Methods

### Patients

Certolizumab-initiated patients were recruited to a prospective observational cohort study, the Biologics in Rheumatoid Arthritis Genetics and Genomics Study Syndicate (BRAGGSS), from 60 UK centres between March 2010 and January 2015.^9^ From the total cohort, 115 patients were selected, according to the following inclusion criteria: RA according to the revised American College of Rheumatology 1987 criteria,^10^ active disease indicated by a 28-joint Disease Activity Score (DAS28) of ≥5.1 despite earlier treatment with at least two non-biologic disease-modifying antirheumatic drugs (nbDMARDs) including methotrexate; patients of Caucasian descent; about to be initiated on subcutaneous certolizumab 400 mg every 4 weeks; baseline visit recorded with ≥1 subsequent visit where serum samples and clinical data were available.

Following initiation of therapy, patients had serum samples collected and disease activity assessed at 3, 6 and 12 months.^9^ Clinical and patient questionnaires, including patient self-reported adherence, were collected at each time point. Adherence was classified as previously defined.^11^ Contributing patients provided written informed consent, and the study was ethically approved (COREC 04/Q1403/37).

### Clinical response

Primary outcome was defined as treatment response at 12 months using European League Against Rheumatism (EULAR) response criteria.^12^ Change in DAS28 C-reactive protein (CRP) (ΔDAS28) was calculated as the difference between the postinitiation and pretreatment DAS28CRP scores. To establish a concentration–effect curve, to determine an optimal drug level cut-off for certolizumab patients with RA, all patients were sorted from high to low drug levels with correlating ΔDAS28, as described previously.^13^

### Measurement of certolizumab drug levels and ADAbs

The measurement of random certolizumab concentrations was performed using a sandwich ELISA and ADAbs using radioimmunoassay (RIA) designed by Sanquin, Amsterdam, The Netherlands, as previously described for infliximab/adalimumab patients.^14^ RIA measures the amount of IgG antibodies specific for certolizumab. Patients were defined as being positive if ADAb titres were >20 AU/mL, as specified by cut-off values generated by Sanquin.

### Statistical analysis

Between-group comparisons were investigated using independent sample t tests, Mann–Whitney U (Wilcoxon) statistics or χ^2^ tests, as appropriate. Ordinal logistic regression was used to test the association between EULAR response at 12 months and drug levels. To assess effect of covariates longitudinally on certolizumab drug and ADAb levels at 3, 6 and 12 months, generalised estimating equation (GEE) with an identity link was used. The multivariate model included significant factors from univariate analysis. The last observation was carried forward for patients with incomplete data. Statistical analyses were performed using STATA for Windows V.13.0 and Graph Pad Prism 6.04 for concentration–effect curve generation.

## Results

### Patients

Over 12 months, 253 serial samples (n=115 patients) were tested for certolizumab drug levels (112 had sufficient sample for ADAb measurement). ADAbs were detected in 37% (42/112 patients) by 12 months of treatment. The baseline characteristics of the total patient population and stratified by detectable ADAbs is shown in table 1. Patients who had detected ADAbs had significantly higher swollen joint counts at baseline. Six patients (14.3%) who developed ADAbs, received a prior biologic versus three patients (4.3%) in the group who did not develop ADAbs. Baseline use and dose of nbDMARDs and oral steroids did not differ significantly between groups (table 1).

**Table 1:** Demographic and clinical characteristics at baseline stratified by antidrug antibody status

Baseline characteristics	Total patient population (n=112)	Patients with antidrug antibodies detected (n=42)	Patients without antidrug antibodies detected (n=70)	p Value
Age years, mean (SD)	58.0±12	57.3±13	58.5±12	0.30
Female (%)	78 (69.6)	27 (64.3)	51 (72.9)	0.30
BMI (IQR)	27.1 (23.4–32.0)	26.6 (23.5–30.9)	27.9 (23.0–33.6)	0.54
*Disease status*				
Disease duration, median (IQR), years	7.0 (3.3–14.4)	8.3 (5.7–15.3)	6.0 (3.3–12.4)	0.095
RF positivity* (%)	61 (73.4)	23 (76.7)	38 (71.7)	0.62
Erosive disease, n*(%)	34 (48.6)	14 (56.0)	20 (44.4)	0.35
DAS28, mean (SD)	5.9 (0.8)	6.0 (0.9)	5.9 (0.8)	0.26
Tender joint count (28 joints), median (IQR)	17 (10–24)	17 (10–24)	17 (11–23)	0.84
Swollen joint count (28 joints), median (IQR)	9 (6–13)	10 (7–16)	8 (6–12)	**0.043**
ESR, median (IQR), mm/h	18 (10.0–37.0)	23.0 (12.0–54.0)	16.5 (10.0–31.0)	0.24
C-reactive protein, median (IQR), mg/L	7.5 (3.3–21.0)	8.9 (3.8–24.0)	7.2 (3.22–20.0)	0.57
Patient global score	75 (56–85)	80 (70–86)	75 (54–82.5)	0.25
Prior biologic (%)	9 (8.0)	6 (14.3)	3 (4.3)	0.059
Oral steroids at baseline (%)*	21 (18.7)	5 (18.5)	16 (19.2)	0.68
*nbDMARD therapy*				
Methotrexate use (%)	60 (53.5)	22 (52.4)	38 (54.3)	0.85
Methotrexate dose, median (IQR) mg/week	20 (15–25)	20 (15–25)	20 (15–25)	0.87
Sulfasalazine (%)	17 (15.2)	6 (14.3)	11 (15.7)	0.84
Sulfasalazine Median (IQR) mg/day	1000 (1000–1000)	1000 (1000–1000)	1000 (1000–1000)	0.75
Leflunomide n (%)	3 (2.7)	1 (2.4)	2 (2.9)	0.88
Hydroxychloroquine (%)	6 (5.3)	2 (4.8)	4 (5.7)	0.83
Baseline nbDMARD use (%)	91 (81.2)	20 (71.4)	71 (84.5)	0.29

*Data for categorical variables is presented as percentage of non-missing data. nbDMARDs listed are the most frequently used in the cohort.

BMI, body mass index; DAS28, Disease Activity Score in 28 joints; ESR, erythrocyte sedimentation rate; nbDMARD, non-biologic disease-modifying antirheumatic drug; RF, rheumatoid factor.

Bold typeface indicates significance at p<0.05.

Of the 20 patients who did not complete 12-month follow-up, 11 (55%) stopped due to inefficacy, 6 (30%) stopped due to adverse events (AEs), 1 (5%) each stopped due to inefficacy/AEs, poor adherence (5%) and imminent heart surgery (5%). Of the patients who experienced AEs, two (one with recurrent chest infections) had drug levels of >40 μg/mL, two had drug levels 28–32 μg/mL (exacerbation of chronic obstructive pulmonary disease symptoms; bilateral pitting oedema and excessive fatigue, respectively), while one patient stopped due to feeling generally unwell with drug levels of 0.2 μg/mL (none with ADAbs). The patient who stopped due to both inefficacy and AEs had drug levels of 2.6 μg/mL and ADAbs of 70 AU/mL at 6 months. Additional information about all patients who developed AEs and their pharmacological tests is provided in online supplementary table S1.

10.1136/annrheumdis-2015-208849.supp1 supplementary table Certolizumab drug-levels and anti-drug antibodies in patients who developed reported adverse events during the study period

### Detection of ADAbs and drug levels

The ADAb titre ranged from 22 to 1600 AU/mL. The presence of ADAbs were significantly associated with lower drug levels over 12 months using GEE (β=−0.037, 95% CI −0.055 to −0.018, p<0.0001), but not independently with 12 months EULAR response (β=0.0013 (95% CI −0.0032, to 0.00061), p=0.18). Using ordinal regression, drug level was associated with 12 months EULAR response (β=0.032 (95% CI 0.0011 to 0.063), p=0.042).

### Concentration–effect curve

**Figure 1:**
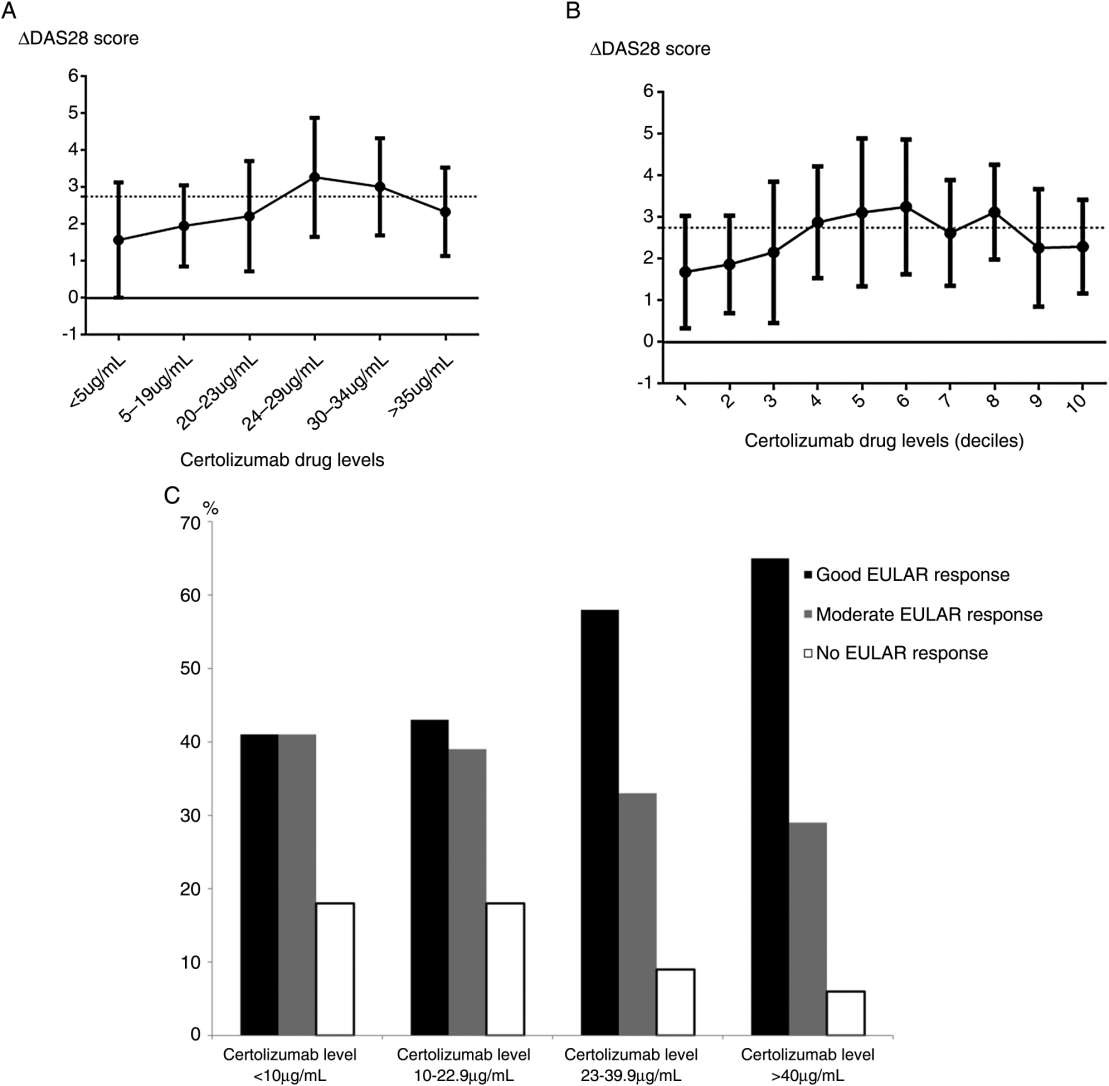
(A) Certolizumab concentration–effect curve at 6 months (using drug-level thresholds). (B) Certolizumab concentration–effect curve at 6 months (deciles). (C) Certolizumab levels in European League Against Rheumatism (EULAR) good, moderate and non-responders at 12 months.

Figure 1A,B shows concentration–effect curves for certolizumab-treated patients. There was a trend for higher certolizumab levels (>23–24 μg/mL) to be associated with improvement in DAS28 from baseline. Patients with the highest certolizumab drug levels had a higher proportion of EULAR good responders at 12 months (figure 1C).

In figures 1A/B each point represents the mean ΔDAS28 (point) and SD (error bars) for the certolizumab drug level range measured at 6 months of treatment stratified in ascending order. Due to the distribution in drug levels, stratification of drug concentrations was performed as described in figure 1A. Figure 1B represents mean ΔDAS28 and SD for deciles generated from the certolizumab drug level data at 6 months, similar to previous studies^13^: (1) 0–10 μg/mL, (2) 11–20 μg/mL, (3) 21–23 μg/mL, (4) 23–24 μg/mL, (5) 25–29 μg/mL, (6) 29–31 μg/mL, (7) 31–33 μg/mL, (8) 31–38 μg/mL, (9) 39–46 μg/mL and (10) >46 μg/mL.

Figure 1C represents the percentages of EULAR responders when stratified by serum certolizumab level. The data represents all 115 patients using last observation carried forward. Patient numbers in each category are as follows: certolizumab levels <10 μg/mL: good EULAR response, 7; moderate EULAR response, 7; no EULAR response, 3; certolizumab levels 10–22.9 μg/mL: good EULAR response, 10; moderate EULAR response, 9; no EULAR response, 4; certolizumab levels 23–39.9 μg/mL: good EULAR response, 34; moderate EULAR response, 19; no EULAR response, 5; certolizumab levels >40 μg/mL: good EULAR response, 11; moderate EULAR response, 5; no EULAR response, 1.

### Baseline and longitudinal factors associated with drug levels and ADAbs

Factors associated with certolizumab drug level in the univariate GEE analysis were gender, adherence, body mass index, CRP and ADAb level (table 2). In the multivariate model after adjustment of confounders, ADAb levels and adherence remained significant (table 2). Certolizumab drug concentrations showed a strong inverse association with certolizumab ADAb level longitudinally over 12 months (table 2).

**Table 2:** Factors associated with drug levels and anticertolizumab antibodies longitudinally over 12 months using GEE

Variable	Certolizumab drug level β coefficient (95% CI)	p Value	ADAb level β coefficient (95% CI)	p Value
*Univariate analysis*				
Age	0.14 (−0.017 to 0.29)	0.08	0.021 (−2.16 to 2.21)	0.99
Female gender*	4.76 (0.21 to 9.29)	**0.040**	−67.36 (−146.14 to 11.41)	0.094
BMI	−0.46 (−0.89 to −0.041)*	**0.032**	−0.92 (−6.03 to 4.19)	0.72
CRP	−0.099 (−0.17 to −0.029)*	**0.005**	0.57 (−0.32 to 1.47)	0.21
ESR	−0.12 (−0.22 to −0.020)*	**0.019**	0.40 (−0.95 to 1.74)	0.58
Baseline methotrexate use	−0.11 (−4.68 to 4.47)	0.96	−3.65 (−53.38 to 46.08)	0.89
Baseline methotrexate dose	0.23 (0.33 to 0.78)	0.43	4.94 (3.74 to −13.6)	0.27
Baseline oral steroid use	3.15 (−1.89 to 8.20)	0.22	−29.77 (−69.54 to 10.0)	0.14
Any nbDMARD use at baseline†	3.15 (−1.89 to 8.20)	0.22	−54.66 (−125.37 to 16.05)	0.14
Antidrug antibody level	−0.037 (−0.055 to −0.018)*	**<0.0001**	–	
Certolizumab drug level	–	–	−2.56 (−4.09 to −1.03)*	**0.001**
Adherence	10.43 (4.76 to 16.11)*	**<0.0001**	−45.05 (−108.35 to 18.25)	0.16
*Multivariate model*‡				
Antidrug antibody level	−0.044 (−0.059 to −0.028)*	**<0.0001**	–	
Adherence	7.08 (0.71 to 13.45)*	**0.029**	–	
Female gender	1.77 (−4.21 to 7.76)	0.56	–	
BMI	−0.13 (−0.68 to 0.43)	0.65	–	
CRP	−0.065 (−0.14 to 0.013)	0.102	–	

*p<0.05.

†nbDMARD use included methotrexate, sulfasalazine, leflunomide or hydroxychloroquine at baseline.

‡Adjusted for variables significant in the univariate analysis. CRP was used instead of ESR in the multivariate model, as was measured in all patients. In the second column, certolizumab drug level is used as the dependant variable in the GEE model; in the fourth column, anticertolizumab antibodies are used as the dependant variable in the GEE model.

ADAb, antidrug antibody; BMI, body mass index; ESR, erythrocyte sedimentation rate; GEE, generalised estimating equation; nbDMARD, non-biologic disease-modifying antirheumatic drug.

## Discussion

This study demonstrates for the first time that certolizumab ADAbs were detectable in 37% of patients with RA over 12 months of treatment. Detectable ADAbs were associated with lower certolizumab drug concentrations, but not independently with treatment response. However, higher certolizumab drug levels were associated with better 12 months EULAR response. Following adjustment, ADAb concentrations and biologic adherence remained the most important predictors of drug levels over time.

Our data demonstrates that even small, non-glycosylated fragments such as certolizumab can be immunogenic. The higher levels of ADAbs detected compared with previous certolizumab trials^1^ ^2^ is noteworthy. However, in contrast to other biologics, ADAbs against certolizumab may be detected more easily even in the presence of drug. Certolizumab is a Fab fragment, monovalent, and therefore, drug–ADAb complexes easily dissociate and can thus be detected despite the drug not necessarily being more immunogenic. RIA, for the detection of ADAbs, has not been used in published certolizumab trials to date. Our data suggests potential for clinical application, but further studies are needed to validate optimal use of the assay in clinical practice. While not being independently associated with treatment response, ADAbs showed a strong inverse correlation with circulating certolizumab drug levels. Therefore, detection of ADAbs need not significantly influence treatment response if sufficient drug is still in circulation but may provide valuable insight into the aetiology of low drug levels in certolizumab-treated patients.

Certolizumab drug concentrations reflect the amount of circulating drug available to bind to the target, so measurement of drug concentrations^9^ may help in determining the aetiology of non-response. This is the first study that has found an association between detectable ADAbs to certolizumab and drug concentrations. Other studies have demonstrated in TNFi treated patients, a high disease burden at baseline may be associated with ADAb formation.^15^ In the current study, pretreatment swollen joint count was significantly higher in patients with detectable ADAbs, supporting data that baseline inflammation may associate with drug concentrations and certolizumab ADAbs.

A strength of our study is the capture of additional factors likely to influence treatment response and drug concentrations in a real-life setting. Of these, adherence was an important factor influencing certolizumab levels (although CIs were wide, as non-adherence was infrequently reported). A low certolizumab drug level in the absence of ADAb formation may prompt a discussion with the patient regarding reasons for non-adherence, also shown to be important in adalimumab-treated patients.^9^ While several TNFi therapeutic drug-monitoring studies have used trough-level serum samples to measure both ADAbs and drug levels to reduce drug interference, ascertainment of random levels is clinically practical. We have previously demonstrated that low random TNFi drug levels in adalimumab-treated patients have been associated with poor treatment response in RA over 12 months.^9^ However, a limitation of our study is that, while 37% of patients had detectable ADAbs to certolizumab, this may under-represent the true value due to drug interference.

Whilst our study did not identify a clear drug concentration cut-off for treatment response in patients with non-trough samples, higher 6 months certolizumab levels (>23–24 μg/mL) were associated with higher ΔDAS28. However, four patients who had an adequate response at 6 and 12 months had undetectable concentration levels, two of whom had ADAbs detected. These patients may represent a unique subset, in which certolizumab could be stopped, avoiding the need for future treatment with expensive biologics.

In summary, certolizumab drug levels may be useful in therapeutic drug monitoring in combination with clinical parameter assessment. Measurement of ADAbs may facilitate the interpretation of low drug levels and provide valuable information about future strategy. More information is required, however, on the cost-effectiveness of using these tests before implementation in clinical practice.

## References

[1] Keystone E, Heijde Dv, Mason D, et al Certolizumab pegol plus methotrexate is significantly more effective than placebo plus methotrexate in active rheumatoid arthritis: findings of a fifty-two-week, phase III, multicenter, randomized, double-blind, placebo-controlled, parallel-group study. Arthritis Rheum 2008;58:3319–29. 10.1002/art.23964 18975346

[2] Smolen J, Landewé RB, Mease P, et al Efficacy and safety of certolizumab pegol plus methotrexate in active rheumatoid arthritis: the RAPID 2 study. A randomised controlled trial. Ann Rheum Dis 2009;68:797–804. 10.1136/ard.2008.101659 19015207 PMC2674556

[3] Garcês S, Demengeot J, Benito-Garcia E The immunogenicity of anti-TNF therapy in immune-mediated inflammatory diseases: a systematic review of the literature with a meta-analysis. Ann Rheum Dis 2013;72:1947–55. 10.1136/annrheumdis-2012-202220 23223420

[4] Vincent FB, Morand EF, Murphy K, et al Antidrug antibodies (ADAb) to tumour necrosis factor (TNF)-specific neutralising agents in chronic inflammatory diseases: a real issue, a clinical perspective. Ann Rheum Dis 2013;72:165–78. 10.1136/annrheumdis-2012-202545 23178294

[5] Kneepkens EL, Plasencia C, Krieckaert CL, et al Golimumab trough levels, antidrug antibodies and clinical response in patients with rheumatoid arthritis treated in daily clinical practice. Ann Rheum Dis 2014;73:2217–9. 10.1136/annrheumdis-2014-205983 25261580

[6] He XH, Shaw PC, Tam SC Reducing the immunogenicity and improving the in vivo activity of trichosanthin by site-directed pegylation. Life Sci 1999;65:355–68. 10.1016/S0024-3205(99)00257-X 10421422

[7] Ganson NJ, Kelly SJ, Scarlett E, et al Control of hyperuricemia in subjects with refractory gout, and induction of antibody against poly(ethylene glycol) (PEG), in a phase I trial of subcutaneous PEGylated urate oxidase. Arthritis Res Ther 2006;8:R12 10.1186/ar1861 16356199 PMC1526556

[8] Colombel JF, Sandborn WJ, Allez M, et al Association between plasma concentrations of certolizumab pegol and endoscopic outcomes of patients with Crohn's disease. Clin Gastroenterol Hepatol 2014;12: 423–31.e1. 10.1016/j.cgh.2013.10.025 24184736

[9] Jani M, Chinoy H, Warren RB, et al Clinical utility of random anti-TNF drug level testing and measurement of anti-drug antibodies on long-term treatment response in rheumatoid arthritis. Arthritis Rheumatol 2015;67:2011–19. 10.1002/art.39169 26109489 PMC4843946

[10] Arnett FC, Edworthy SM, Bloch DA, et al The American Rheumatism Association 1987 revised criteria for the classification of rheumatoid arthritis. Arthritis Rheum 1988;31:315–24. 10.1002/art.1780310302 3358796

[11] Bluett J, Morgan C, Thurston L, et al Impact of inadequate adherence on response to subcutaneously administered anti-tumour necrosis factor drugs: results from the Biologics in Rheumatoid Arthritis Genetics and Genomics Study Syndicate cohort. Rheumatology (Oxford) 2015;54:494–9. 10.1093/rheumatology/keu358 25213131 PMC4334684

[12] van Gestel AM, Prevoo ML, van't Hof MA, et al Development and validation of the European League Against Rheumatism response criteria for rheumatoid arthritis. Comparison with the preliminary American College of Rheumatology and the World Health Organization/International League Against Rheumatism Criteria. Arthritis Rheum 1996;39:34–40. 8546736 10.1002/art.1780390105

[13] Pouw MF, Krieckaert CL, Nurmohamed MT, et al Key findings towards optimising adalimumab treatment: the concentration-effect curve. Ann Rheum Dis 2015;74:513–8. 10.1136/annrheumdis-2013-204172 24326008

[14] Rispens T, de Vrieze H, de Groot E, et al Antibodies to constant domains of therapeutic monoclonal antibodies: anti-hinge antibodies in immunogenicity testing. J Immunol Methods 2012;375:93–9. 10.1016/j.jim.2011.09.011 21986105

[15] Bartelds GM, Krieckaert CL, Nurmohamed MT, et al Development of antidrug antibodies against adalimumab and association with disease activity and treatment failure during long-term follow-up. JAMA 2011;305:1460–8. 10.1001/jama.2011.406 21486979

