# Supplementary material for: High frequency of antidrug antibodies and association of random drug levels with efficacy in certolizumab pegol-treated patients with rheumatoid arthritis: results from the BRAGGSS cohort
Source: Ann Rheum Dis. 2016 May 31;76(1):208–13. doi: 10.1136/annrheumdis-2015-208849 (PMC5264213; doi:10.1136/annrheumdis-2015-208849)
Supplement: supplementary table — Certolizumab drug-levels and anti-drug antibodies in patients who developed reported adverse events during the study period [file annrheumdis-2015-208849supp_table.pdf]

Supplementary Table: Certolizumab drug-levels and anti-drug antibodies in patients who developed reported adverse events during the study period

Patient	Reported adverse event details	Certolizumab drug level (µg/mL)	ADAb level AU/ml
**<u>Respiratory</u>**			
1	Sepsis requiring admission to hospital	65	0
2	Pneumonia	62	0
3	Chest infection	28	38
4*	Chest infection	46	0
5*	Chest infection (+inefficacy)	2.6	70
6*	Exacerbation of Chronic Obstructive Airways Disease symptoms	28	0
7	Chest symptoms- increased dyspnoea	0.4	1600
8	Pneumonia	4.3	500
9	Chest infection	35	0
10	Chest infection/possible progression of lung fibrosis	36	0
**<u>Urinary Tract</u>**			
11	Urinary tract infection	94	0
12	Urinary tract infection	49	0
13	Urinary tract infection	34	0
14	Recurrent urinary tract infections	26	0
15	Recurrent urinary tract infections	54	0
**<u>Miscellaneous</u>**			
16	Irregular heart rate, lethargy, unwell	0	190
17	Rash over face	10	640
18	Flu –like symptoms	25	210
19	Recurrent eye cysts	41	0
20	Sore throat (leading to temporary discontinuation)	35	0
21*	Patient reported unwell	0.2	0
22	Reported adverse event (no details)	16	35
23*	Reported adverse event (no details)	44	2
24*	Bilateral oedema and fatigue	32	0

Whilst certolizumab drug levels were not performed at the time of the event, the results of the closest serum sample to the date of the adverse event are reported above. Whilst the majority of above events led to temporary discontinuation, those marked with (*) led to permanent discontinuation.
